
JOURNAL INFORMATION
==============================
NLM Title Abbreviation: Med Chir Rev
NLM Journal ID: 0034617

The Medico-Chirurgical Review

Publisher: Biomedical Journal Digitization Project

ARTICLE INFORMATION
==============================
PMCID: PMC4966255
PMID: 29917626
Article version: 1
Subjects: Reviews, Diseases Produced by Malaria

Memoir on Lateral Depression of the Thoracic Parietes

Print publication date: 1828 Oct 1
Volume: 9
Issue: 18
First page: 311
Last page: 312
1828 Jul
License: This work is free of known copyright restrictions. For more information, please see PMC Back Issue Digitization.
License URL: https://creativecommons.org/publicdomain/mark/1.0/

==============================

### III.

### *Memoir on Lateral Depression of the Thoracic Parietes.* By Baron Dupuytren.

This is not the contraction or depression described by Laennec, the result of inflammation and effusion in one side of the chest—but a congenital deformity of no unfrequent occurrence, and one which exercises a considerable influence on the functions of the heart and lungs. Not a month passes without numerous examples of this congenital defect being presented to the observation of Baron Dupuytren. It consists in a greater or lesser depression of one side of the thorax, with a proportionate protuberance or saliency of the sternum—the abdomen forwards—or the vertebral column backwards. There are a few scattered observations on this species of deformity, in the writings of Van Swieten, Petit, and others, who have attributed it to rickets, or other diseases, and who were evidently unacquainted with its nature, causes, effects, or remedy. This deformity is seen more especially among the children of scrofulous, rickety, and unhealthy parents, residing in damp and unwholesome localities, and who are badly fed and scantily clothed, In children affected with this deformity, the ribs of the two sides are sometimes so much approximated, that the hand can span across the chest, the sternum being, of course, bulged out in proportion; for whatever space is lost in one direction must necessarily be gained in another. But this equipoise of dimensions does not enable the heart and lungs to perform their functions as well as in a properly formed thorax. There is caused an habitual shortness of breath and voice—a great oppression—a sense of indiscribable anxiety. In the newly-born infant there is great difficulty in sucking, threatening of suffocation, and a necessity for frequently dropping the nipple, which sets the infant crying. At a more advanced age, the voice is broken, and the sentences rendered very short. All these symptoms are much increased by exercise, especially by ascending stairs, or a hill—as in people who have organic diseases of the heart. The resemblance is farther increased by irregularities in the pulse, and other symptoms attendant on cardiac diseases. While asleep, people with this deformity generally breathe with their mouths open, and make much noise. Their sleep too is much broken by terrific dreams. These symptoms are sometimes carried to the pitch of impeding the development of the vital functions, and death is the consequence almost immediately on birth—or at various periods afterwards, according to the degree of deformity. Even when death is not thus produced, the infants are often seen lingering out in a sickly emaciated condition, with a very feeble development of their intellectual faculties. It is very remarkable, that a frequent attendant on this vice of conformation, is a considerable swelling of the tonsils—a consequence or connexion for which we cannot account. This swelling of the tonsils adds greatly to the difficulty of breathing, and other inconveniences produced by the lateral depression. M. Dupuytren has been frequently obliged to remove these tonsils on account of their great enlargement, and the embarrassment which they occasioned the respiration. This operation always gave much relief. Pulmonary catarrh is another frequent attendant on this deformity, and proves a very formidable complication, especially where the tonsils are enlarged. The breathing is then incommoded by three different causes. But the most dangerous affection which can supervene on this deformity, is hooping-cough. An infant with lateral depression, enlarged tonsils, and hooping-cough, presents one of the most melancholy spectacles of suffering that can be imagined! He generally dies in one of the paroxysms of coughing. Baron Dupuytren has been obliged to remove the tonsils of children at the breast, rather than see them perish by suffocation. The operation, at such an early period of life, is extremely difficult, and nothing but the most urgent circumstances would induce him to attempt it. One of his most ingenious pupils, however, Doctor Lemaitre, has invented a speculum oris, which renders the operation comparatively easy and void of danger. By means of this instrument, the mouth may be kept open, the tongue depressed and motionless, and the tonsils readily removed.*

An examination of many children who had died of this disease, or of it and some of its complications, demonstrated a retardation of development in the skeleton, as evinced by various phenomena, such as the non-closure of sutures in the head, the persistance of epiphyses, the softening of cylindrical bones, &c. The lungs were found, of course, to take the impressions of the different parts of the thorax.

*Treatment.* This, at first sight, might seem hopeless—but it is not so. Much may be done by bitters, tonics, and those remedies which are employed in scrofula, care being taken not to push them so far as to render the respiration incommoded. To these general means, must be joined local ones; and the most important is exercise, (especially of those muscles which extend from the chest to the arms and shoulders,) combined with frequent pressure on the sternum. The exercise has for its object to elevate the ribs, and consequently to expand the dimensions of the thorax. Any person may invent a simple contrivance for executing this kind of exercise by means of pulleys, cords, and weights. With this exercise, as we before observed, the sternum ought to be daily pressed backwards, so as to bring the chest to its natural rounded form, instead of its flattened shape. By these means the Baron has effected numerous cures of this vice of conformation, when the cases were thought to be totally beyond the reach of art. This pressure is better effected by the hand than by any spring or other apparatus. The Baron places the back of the little patient against his knee, or against a wall, and then makes pressure on the sternum with the palm of his hand, removing the pressure at every inspiration, and re-applying at each expiration. "I have often (says he) observed with attentive curiosity the immediate effects of this exercise—viz. a momentary return of the chest to its cylindrical form, with a corresponding freedom of breathing—and a relapse into its ordinary flattened shape when the pressure was removed." This pressure should be made from ten to a hundred times a day, if possible, and should be continued for several minutes each time. The oftener the pressure is exercised, and the longer it is continued at a time, the more efficacious it will be found. The mother is generally the only one who should be entrusted with this operation. No other will take the pains, or have the patience to persevere. Four cases are detailed in illustration of the foregoing descriptions and precepts; but they do not require insertion in this place, as the directions are plain and intelligible. We think it is the duty of every medical practitioner to carefully examine the naked bodies of infants that appear to labour under any difficulties of function about the chest. By this early inspection much error may be avoided—much good may be done.—Repertoire.

* We wonder that some of our English "voyageurs" do not send over this instrument to England. It must prove extremely useful on many occasions, while surgeons are operating on parts within the mouth or throat.—Ed.
